# Determinants of facilitated health insurance enrollment for patients with HIV disease, and impact of insurance enrollment on targeted health outcomes

**DOI:** 10.1186/s12879-018-3035-7

**Published:** 2018-03-16

**Authors:** Renae Furl, Shinobu Watanabe-Galloway, Elizabeth Lyden, Susan Swindells

**Affiliations:** 1 Department of Internal Medicine, Division of Infectious Diseases, University of Nebraska Medical Center, 988106 Nebraska Medical Center, Omaha, NE 68198-8106 USA; 2 College of Public Health – Epidemiology, Maurer Center for Public Health 3023, University of Nebraska Medical Center, Omaha, NE 68198-4395 USA; 3 College of Public Health – Biostatistics, Maurer Center for Public Health 3046, University of Nebraska Medical Center, Omaha, NE 68198-4375 USA

**Keywords:** HIV infection, Affordable care act, AIDS drug assistance program, Health outcomes, And health disparities

## Abstract

**Background:**

The introduction of the Affordable Care Act (ACA) has provided unprecedented opportunities for uninsured people with HIV infection to access health insurance, and to examine the impact of this change in access. AIDS Drug Assistance Programs (ADAPs) have been directed to pursue uninsured individuals to enroll in the ACA as both a cost-saving strategy and to increase patient access to care. We evaluated the impact of ADAP-facilitated health insurance enrollment on health outcomes, and demographic and clinical factors that influenced whether or not eligible patients enrolled.

**Methods:**

During the inaugural open enrollment period for the ACA, 284 Nebraska ADAP recipients were offered insurance enrollment; 139 enrolled and 145 did not. Comparisons were conducted and multivariate models were developed considering factors associated with enrollment and differences between the insured and uninsured groups.

**Results:**

Insurance enrollment was associated with improved health outcomes after controlling for other variables, and included a significant association with undetectable viremia, a key indicator of treatment success (*p* < .0001). We found that minority populations and unstably housed individuals were at increased risk to not enroll in insurance.

**Conclusion:**

The National HIV/AIDS Strategy calls for new interventions to improve HIV health outcomes for disproportionately impacted populations. This study provides evidence to prioritize future ADAP-facilitated insurance enrollment strategies to reach minority populations and unstably housed individuals.

## Background

Improved health outcomes have been documented for individuals with HIV disease with access to health insurance, including sustained viral suppression (a key indicator of treatment success) [1], less frequent and shorter hospital stays [2], and decreased mortality [3]. However, HIV-infected persons have lower rates of insurance coverage than other groups with just 17% having private insurance coverage [4], compared to 63.9% of Americans in 2012 [5]. Such a striking contrast in insurance coverage is not surprising, given that many risk factors for HIV acquisition are the same as those for being uninsured. Minority populations, individuals living in poverty and young persons are greatly overrepresented in the US HIV epidemic and among the uninsured [6–11].

Ryan White HIV/AIDS Programs (RWHAP) are a safety net available to assist with the costs of HIV care and treatment for uninsured and underinsured individuals. AIDS Drug Assistance Programs (ADAPs) are available in each of the U.S. states and territories to provide anti-HIV medications, and serve about one third of people in care for HIV nationally [12]. Historically, ADAPs have implemented health insurance continuation and purchasing as a cost-saving strategy [13], but an additional benefit is that insurance access enhances access to medical care. The purchase of medications by ADAP could be seen to promote drug adherence, but the purchase of insurance could promote care adherence.

Despite the availability effective antiretroviral therapy, there are still HIV-infected patients not accessing care as illustrated by the “HIV Care Continuum”. The continuum is a monitoring device designed to illustrate the steps in HIV Care, and demonstrates that in the U.S., 14% of HIV-infected persons are not diagnosed, of those diagnosed 80% are in care but only 40% are retained in care, and only 30% achieve viral suppression [14]. The continuum also illustrates the reinforcing cycle wherein the same population groups with poor HIV-related health outcomes are those with higher rates of new HIV infections, including minority populations and youth [15, 16]. This harmful cycle has made improving HIV care and medication adherence top priorities of National HIV/AIDS Strategy, with a focus on disproportionately impacted populations [17].

At the initiation of the Affordable Care Act (ACA), ADAPs were directed to “vigorously pursue” uninsured participants to enroll in health insurance plans and to assist with the resulting costs as able [18]. In response to this directive, the Nebraska ADAP coordinated a statewide insurance enrollment strategy, offering assistance with insurance premiums, medication copayments, and outpatient cost-sharing up to $5000 per ADAP recipient per year. This activity also presented an unprecedented opportunity to understand whether ADAP-facilitated health insurance enrollment could improve health outcomes.

The objectives of the study were to identify demographic and clinical factors associated with ADAP-facilitated insurance enrollment in Nebraska, and compare health outcomes between participants who enrolled in insurance and those who did not.

## Methods

### Study design and participants

This was a retrospective cohort study of ADAP participants recruited for insurance enrollment, who also receive medical care at the University of Nebraska Medical Center (UNMC). All participants were uninsured adults greater than 18 years of age, who met the ADAP eligibility guidelines and the ACA eligibility guidelines during the first open insurance enrollment period from October 1, 2013 to March 31, 2014. The ADAP guidelines included being a Nebraska resident with HIV infection, having income less than 200% federal poverty level, and not being eligible for other insurance coverage. The additional ACA eligibility guidelines included being a citizen or lawfully present in the U.S., and not being incarcerated.

Nebraska ADAP developed a tiered ACA enrollment strategy with input from stakeholders across the state. This stakeholder team prioritized enrollment outreach dates by patient groups to maximize program savings. Potentially eligible individuals were identified and grouped based on data variables tracked for ADAP eligibility reporting. Individuals earning greater than 100% of the federal poverty level (FPL) conferred the largest costs savings to the program because they were eligible for premium tax credits, lowering their health insurance premiums. Enrollment outreach to this ***primary group*** started on October 1, 2013. Because Nebraska opted not to expand Medicaid coverage to persons earning less than 100% FPL, stakeholders elected to also enroll these individuals in insurance. Expecting these resulting premiums to be more costly because no tax credits would be applied, individuals were screened by the clinic pharmacist for an 80% timely refill benchmark, in order to ensure program cost-savings. ADAPs must demonstrate cost-savings in the aggregate to pay insurance premiums as opposed to medications. Outreach to the ***cost-saving group*** started on February 21, 2014. The final group, the ***“churn” group***, was targeted throughout the enrollment period and was identified through ongoing case management assessments. “Churning” refers to fluctuations in patient income which impact program and insurance eligibility, putting patients at risk of coverage or treatment interruptions (Sommers & Rosenbaum, 2011). Program case managers were prepared to reassess patient situations, to avoid gaps caused by churning. Individuals in all three of the enrollment groups were contacted by mail, phone, and in person by the ACA enrollment team.

### Data sources and collection methods

Study participants were followed for one year, referred to as the participants’ coverage year. The coverage year start date was staggered for the insured group based on the insurance enrollment date, and ranged from January 1, 2014 to May 1, 2014. For the not insured group, January 1, 2014 was used as the coverage year start date. For the small number of participants who reported moving during their coverage year, health outcome and drug adherence measures only included the time period before this move.

Data were collected from three sources including the UNMC Electronic Medical Record (EMR), the Organ Transplant Tracking Registry (OTTR) database, and the ScriptGuideRx pharmacy benefit management database (SGRx). The EMR was utilized to capture clinical variables, diagnosis of comorbidities and other measures. Clinical outcomes were captured at the last visit in the participant’s coverage year to maximize potential differences between the intervention and control groups. Three common comorbidities were selected for comparison: diabetes mellitus, hyperlipidemia and hypertension. All are common in persons with chronic HIV disease, and also have metrics for diagnosis and management that are readily available in the EMR [19]. For participants who failed to attend any UNMC HIV clinic visits, monitoring values were only recorded if the patient was hospitalized or seen by other providers. Based on previous studies, patients for whom viral load levels were not drawn during the coverage year were included in the “not virally suppressed” category. Viral suppression was defined as a last HIV-1 RNA level < 20 copies/mL. The OTTR database is utilized by ADAP staff to meet Health Resources and Services Administration reporting requirements associated with Ryan White program funding. OTTR was utilized for this study to collect demographic and other HIV related variables (i.e. HIV diagnosis date and transmission risk). The SGRx database was utilized to collect pharmacy claims information including drug names, number of pills supplied and refill dates. These data allowed calculation of an HIV medication refill percentage, based on the methodology for the Proportion of Days Covered (PDC), endorsed by the Pharmacy Quality Alliance [20]. The PDC is calculated by dividing the coverage days accounted for by the patient’s refill records, by days in the coverage year.

### Statistical analysis

Descriptive statistics were used to summarize demographic characteristics, comorbidities, and clinical measurements of the study population. Baseline demographic and clinical information were compared between two the groups (those who enrolled in insurance and those who did not) using the chi-square test, Fisher’s exact test or student’s t-test as appropriate. A univariate analysis was conducted to examine associations between the baseline factors and significant outcome measures including obtaining undetectable viral load, PDC refill ratio, number of other clinic visits and failed clinic appointments. Variables significant in univariate analysis were included in multivariate models to examine the independent effect of insurance enrollment on HIV-related and primary health outcomes. Logistic regression or multiple linear regression was used as appropriate for the distribution of the outcome measures. All tests were 2-sided and *p*-value < 0.05 were considered statistically significant. SAS software version 9.3 (SAS Institute, Cary North Carolina), was utilized to carry out all statistical analysis.

This study was approved by the Institutional Review Board at UNMC.

## Results

**Fig. 1:**
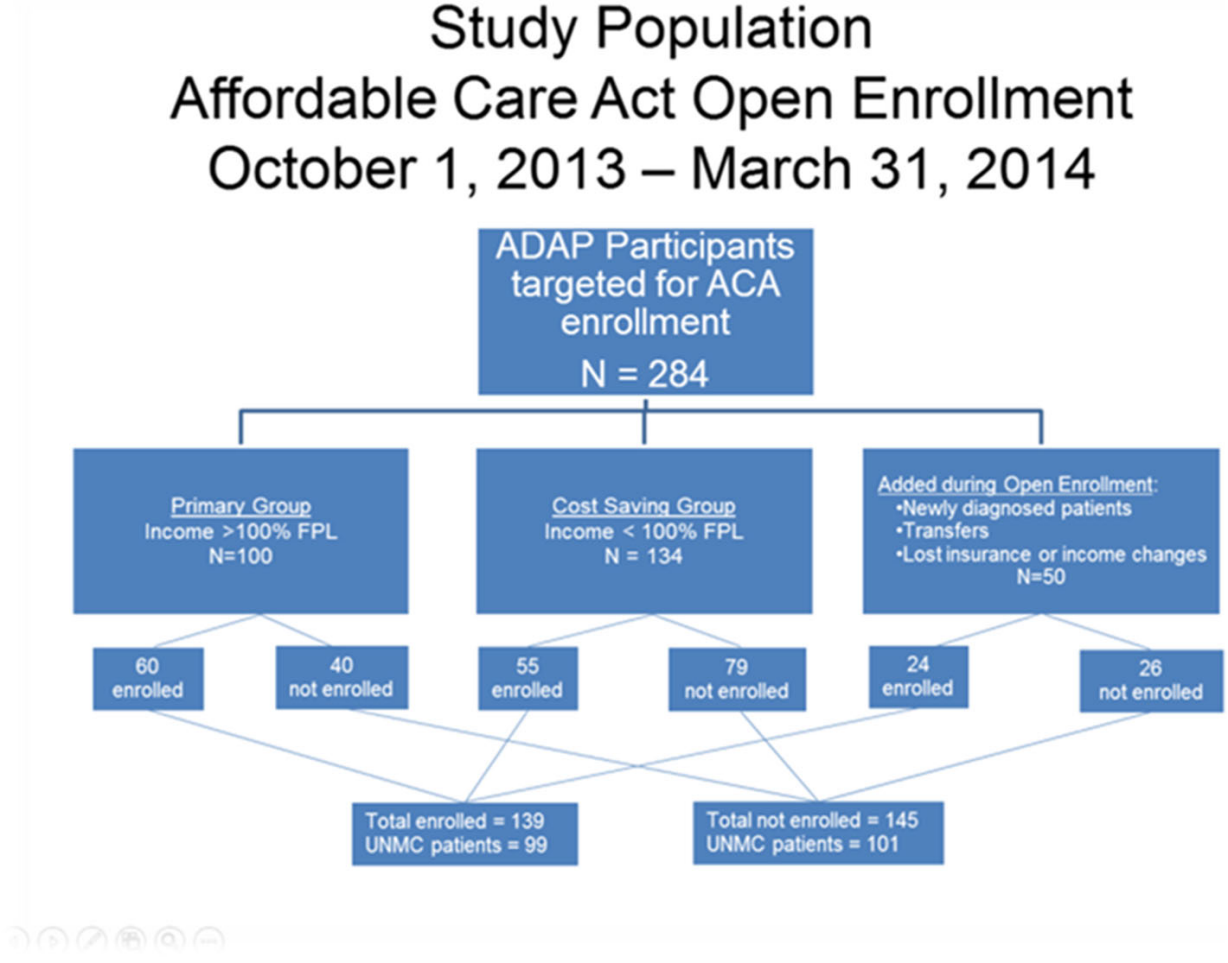
Study Population Affordable Care Act Enrollment October 1, 2013 – March 31, 2014

From October 1, 2013 to March 31, 2014, Nebraska ADAP identified 284 total participants who were eligible for the ACA (see Fig. 1). There were 100 participants in the primary group, 134 in the cost-saving group and 50 in the churn group. The enrollment numbers and rates amongst these enrollment groups were 60 (60%), 55 (41%) and 24 (48%) respectively. Of the total 139 participants who enrolled in insurance, 99 receive medical care at UNMC and represent the intervention group. Of the total 145 participants who did not enroll in insurance, 101 receive medical care at UNMC and represent the comparison group.

Demographic and clinical features for the 200 patients eligible for insurance enrollment are outlined in Table 1. The groups who enrolled in health insurance and those who did not were compared. Age was significantly related to insurance enrollment, with an average age of 44.2 years for the insured group compared to 40.6 years in the uninsured group (*p* = .018). No gender differences were observed. Racial and ethnic backgrounds of participants influenced the likelihood of insurance enrollment (*p* = .0024). Among non-Hispanic white individuals recruited for enrollment, 61.7% enrolled in insurance coverage, in contrast to non-Hispanic black individuals of whom 37.7% enrolled and 62.3% did not. For Hispanic individuals, only 33.3% enrolled and 66.7% did not. Unstable housing contributed to insurance enrollment (*p* = .014), with only 21.1% of unstably housed individuals enrolling compared to 52.1% of stably housed individuals.

**Table 1:** Demographic and baseline clinical characteristics of patients enrolled in insurance through the AIDS Drug Assistance Program vs. not insured

	Insured (*n* = 99)		Not Insured (*n* = 101)		
	Number	%	Number	%	*p*-value
Age					
20–24 years of age	2	2.0%	7	7.0%	.057
25–34 years of age	22	22.2%	23	22.8%	
35–44 years of age	24	24.2%	31	30.7%	
45–54 years of age	29	29.3%	31	30.7%	
55–69 years of age	22	22.2%	9	8.9%	
Mean and standard deviation	44.23	10.9	40.62	10.5	.018
Gender					
Male	75	75.8%	71	70.3%	.71
Female	23	23.2%	29	28.7%	
Transgender M-F	1	1.0%	1	1.0%	
Race/Ethnicity					
Non-Hispanic White	58	58.6%	36	35.6%	.0024
Non-Hispanic Black	26	26.3%	43	42.6%	
Hispanic any race	10	10.1%	20	19.8%	
Other race	5	5.1%	2	2.0%	
Marital status					
Single	80	80.8%	71	70.3%	.22
Married	8	8.1%	13	12.9%	
Other	11	11.1%	17	16.8%	
Housing Status					
Stable	95	96.0%	86	85.1%	.014
Unstable	4	4.0%	15	14.9%	
Employment Status					
Not working	31	31.3%	44	43.6%	.086
Working part time	30	30.3%	32	31.7%	
Working full time	38	38.4%	25	24.8%	
Income					
Below 100% FPL	56	56.6%	63	62.4%	.40
100%–200% FPL	43	43.4%	38	37.6%	
Household size					
One	86	86.9%	82	81.2%	.22
Two	6	6.1%	7	6.9%	
Three or more	7	7.1%	12	11.9%	
Time since HIV diagnosis (yrs)					
< 5 years since diagnosis	24	24.2%	30	29.7%	.53
5–9 years since diagnosis	23	23.2%	30	29.7%	
10–14 years since diagnosis	24	24.2%	20	19.8%	
15–19 years since diagnosis	14	14.1%	12	11.8%	
> 20 years since diagnosis	14	14.1%	9	8.9%	
Mean and standard deviation	10.96	7.4	9.20	6.7	.079
HIV transmission risk					
Men who have sex with men	57	57.6%	39	38.6%	.022
Heterosexual contact	33	33.3%	52	51.5%	
Other	9	9.1%	10	9.9%	
AIDS diagnosis?					
Yes	45	45.5%	41	40.6%	.49
No	54	54.5%	60	59.4%	
History of drug abuse?					
Yes	17	17.2%	25	24.8%	.19
No	82	82.8%	76	75.2%	
History Alcohol abuse?					
Yes	12	12.1%	14	13.9%	.71
No	87	87.9%	87	86.1%	
History mental illness?					
Yes	33	33.3%	34	33.7%	.96
No	66	66.7%	67	66.3%	

Race/ethnicity collapsed into the category “other” including Alaskan Native, American Indian, Asian, Multiracial, Native Hawaiian Pacific Islander

Among the other predictors of insurance enrollment, one surprising result was that men who have sex with men (MSM) were much more likely to enroll in insurance than all other HIV transmission risk groups, with 57.6% of insurance enrollees reporting MSM transmission risk versus just 33.3% of insurance enrollees reporting heterosexual transmission risk (*p* = .022). Subsequent investigation revealed an association between race/ethnicity and HIV transmission risk variable, with the majority of black/non-Hispanic individuals reporting heterosexual activity as their HIV transmission risk (41 of 69, 59.4%), compared to just 31 of 94 white/non-Hispanic individuals (33.0%), and 13 of 37 Hispanic/other individuals (35.1%, *p* = .006). No difference in insurance enrollment was seen based on history of drug or alcohol use (*p* = .19 and *p* = .71), or history of mental illness (*p* = .96).

Insurance enrollment predictor variables with a *p*-value < 0.1 were considered for the multivariate analysis. The final model for the logistic regression utilized race, age and housing variables, and selected race and housing status as the best predictors of insurance enrollment. Unstably housed individuals were 4.9 times less likely to obtain insurance than stably housed individuals (*p* = .0082, data not shown). Black/non-Hispanic individuals had 2.5 times the odds of not enrolling in insurance, (*p* = .0072), and Hispanic/other individuals had 2.3 odds of not enrolling in insurance, compared to white/non-Hispanic individuals (*p* = .043).

There were significant differences between the insurance groups regarding HIV-related health outcome measures (see Table 2). A comparison of the PDC refill ratio showed a significant difference between the groups with an average HIV medication coverage rate 17.2% higher among the insured group (*p* < .0001). Individuals in the uninsured group have free access to medications through ADAP, so access to HIV medications was not a factor. Participants in the insured group averaged 2.4 HIV clinic visits over their coverage year, compared to 1.8 visits in the uninsured group (*p* = .0001). No differences were observed in CD4 cell counts, or in measurements of blood pressure and BMI. Insurance status was associated with viral suppression (*p* < .0001), with 58.7% of virally suppressed individuals belonging to the insurance group compared to 41.3% belonging to the uninsured group.

**Table 2:** Health outcome comparisons among Insured AIDS Drug Assistance Program participants vs Not Insured

	Insured (n = 99)		Not Insured (n = 101)		
	Mean	SD	Mean	SD	*p*-value
PDC medication refill ratio/yr	78.65	22.18	61.45	36.12	<.0001
Total count HIV clinic visits	2.36	1.11	1.75	1.08	.0001
Total count medications	5.64	4.74	4.08	3.61	.0096
Total count HIV medications	1.98	1.15	1.69	1.25	.093
Lab values					
CD4 count	701.42	360.69	615.52	384.29	.13
Low-density lipoprotein	100.40	35.54	92.29	29.84	.19
Diastolic blood pressure	127.20	16.49	130.10	20.89	.28
Systolic blood pressure	80.57	9.28	79.94	9.34	.65
Body Mass Index	26.95	8.56	26.27	5.87	.54
	Number	%	Number	%	*p*-value
HIV viral load undetectable?					
Yes	84	84.8%	59	58.4%	<.0001
No	15	15.2%	42	41.6%	
Accessed other type doctors?					
Yes	61	61.6%	32	31.7%	<.0001
No	38	38.4%	69	68.3%	
“No Shows” in HIV clinic?					
Yes (one or more)	32	32.3%	52	51.5%	.0060
No	67	67.7%	49	48.5%	
ER Visits?					
Yes	14	14.1%	14	13.9%	.95
No	85	85.9%	87	86.1%	
Hospitalized?					
Yes	5	5.1%	9	8.9%	.41
No	94	94.9%	92	91.1%	
Diabetes Mellitus diagnosis?					
Yes	6	6.1%	4	4.0%	.75
No	93	93.9%	86	85.4%	
Hyperlipidemia diagnosis?					
Yes	25	25.2%	9	8.9%	.0064
No	74	74.7%	81	80.2%	
Hypertension diagnosis?					
Yes	21	21.2%	18	17.8%	.84
No	78	78.8%	72	71.3%	

*Abbreviation* *PDC* Proportion Days Covered, calculated by dividing the coverage days accounted for by the patient’s refill records, by the days in the coverage year

Outcomes designed to measure access to primary health care for conditions other than HIV disease included total medication counts and total number of visits with other types of physicians. Both showed greater utilization among the insured group. The insured participants averaged 5.6 medications per patient, compared to 4.1 among the uninsured group (*p* = .0096). Among 93 patients who were seen by other types of physicians, 61.6% belonged to the insurance group, compared to 31.7% from the uninsured group (*p* < .0001). Failed HIV clinic appointments were more common among the uninsured group (32.3% vs 51.5%, *p* = .006). Frequency of emergency room visits and hospitalizations were similar between the groups. There was no difference in rates of diagnosis of diabetes or hypertension, but insured persons were more likely to be diagnosed with hyperlipidemia (*p* = .0064).

Health outcome variables that were significant in the univariate analysis were individually compared to the predictors of insurance enrollment with a *p*-value < 0.1, to select the final model. The only variables that met these criteria were age and race (see Table 3). Among the continuous health outcome measures of PDC refill ratio and total HIV clinic visits per year, belonging to the insurance group remained the most significant predictor of having a higher refill ratio and more clinic visits (β = 17.02, *p* = .0002 and β = .64, *p* < .0001). With regard to the outcome of total medication count, age and insurance group were significant predictors (β = .14, *p* < .0001 and β = 1.18, *p* = .044).

**Table 3:** Multivariable linear regression analysis on adherence & clinical outcomes

	Refill Ratio/yr		HIV Clinic visits/yr		Total count meds	
	β	*p*-value	β	*p*-value	β	*p*-value
Insurance Group	17.023	.0002	.64	<.0001	1.18	.044
Race/Ethnicity						
Hispanic/Other	–	–	.59	.0062	–	–
Age	–	–	.017	.022	.14	<.0001

The multiple logistic regression model showed that belonging to the insurance group was the most significant predictor of obtaining an undetectable viral load, or not failing an HIV clinic appointment, after adjusting for age and race (see Table 4). The odds of not having an undetectable viral load were 4.0 times higher in the uninsured group compared to the insurance group (*p* < .0001). The odds were 1.9 times higher in the uninsured group to fail an appointment the HIV clinic (*p* = .04), and non-Hispanic black participants were at two times greater odds of failing appointments (*p* = .046). Participants had 3.4 times the odds of accessing other physicians if they belonged to the insurance group (*p* = .0001), compared to the uninsured group.

**Table 4:** Multivariable Logistic Regression analysis on clinical outcomes

	Viral Load Undetectable?		No Shows?		Other Clinic Access?		Hyperlipidemia?	
	Odds Ratio (95%CI)	*p*-value	Odds Ratio (95%CI)	*p*-value	OR (95%CI)	p-value	Odds Ratio (95%CI)	*p*-value
Insurance Group	4.0 (2.0–8.0)	<.0001	1.9 (1.0–3.4)	.040	3.4 (1.8–6.3)	.0001	2.5 (1.0–6.3)	.049
Race /Ethnicity Non-Hispanic Black	–	–	2.0 (1.0–3.9)	.046	–	–	–	–
Age	–	–	–	–	.95 (.92–.98)	.0010	.89 (.84–.93)	<.0001

Confidence Interval is abbreviated CI

## Discussion

Enrolling in ADAP-sponsored health insurance conferred several important direct benefits. Insured persons were more likely to have undetectable HIV viremia, and more likely to have access to non-HIV providers. Having well controlled HIV disease, as evidenced by undetectable viremia, is a key indicator of successful therapy and is strongly associated with improved health outcomes, and decreased risk of transmission to others [21]. Our study also showed that factors that impacted enrollment in insurance are similar to those that put persons at high risk for HIV infection, and at high risk for poor HIV health outcomes, namely younger age and being from a minority population. We have used these findings to target those at risk for not enrolling in ADAP-sponsored health insurance during subsequent enrollment periods.

Differences between the insurance groups were not surprising due to several considerations. Patients actively engaged in routine care likely attended a clinic appointment during the six month ACA open enrollment period, connecting them with a case manager who could answer questions and encourage insurance enrollment. Often these individuals could be enrolled before leaving their clinic appointment. Although the number of unstably housed individuals was small, the significant impact of unstable housing in this study, as a predictor of insurance non-enrollment, reflects a large body of data implicating unstable housing as a top predictor of poor outcomes along the entire HIV Care Continuum [22].

Consideration of the three enrollment groups, defined by income above or below the federal poverty level, was not accounted for in the analysis despite these groups being targeted for different lengths of time. This omission was made after careful consideration. The churn group was categorized based on real time changes in income. Such changes would equally impact low or high income individuals. For example, an individual could join the churn group from an increase in income resulting in disqualification from Medicaid, or an individual could join the churn group from a loss of employment/income. With regard to the cost-saving group, these individuals were screened with an 80% timely refill benchmark to ensure overall program cost-savings. Therefore, although the cost-saving group had a shorter enrollment outreach window, any bias resulting from lower income/shorter outreach period was thought to be mitigated based on the timely refill requirement in this group. In spite of the timely refill requirement for this subset, there was still a striking difference with regard to PDC refill ratio and viral suppression for the insurance group.

The results of this study represent one Nebraska ADAP population accessing care at UNMC, and the population was small and may not apply to other groups. Since this study was conducted, research considering 3933 participants enrolled in Virginia ADAP also revealed an association between insurance enrollment and HIV viral suppression (*p* < .001) [23]. Virginia enrolled 47.1% of ADAP participants in insurance coverage, compared to 48.9% of the Nebraska ADAP population, 49.5% of the UNMC population. These figures demonstrate similar enrollment success considering different programs. Future comparisons between states are planned.

In addition to the small study population, other study limitations included the fact that we could not account for participants who moved or changed providers without notifying program staff. Also, designation in the insurance group or the uninsured group remained the same for the entire coverage year even when participants could have moved to other coverage. Finally, we cannot exclude possible selection bias prompting patients who were more concerned about their health and more adherent to therapy from being more likely to accept insurance.

The complex nature of insurance and the ACA, especially for individuals who have potentially never had private insurance coverage in the past, could have created a barrier to enrollment. A study conducted at the UNMC HIV clinic just before the first ACA open enrollment period found that of the 406 survey respondents, only 21% reported that they felt they had or will eventually benefit from the ACA and 57% reported they did not believe that they are informed enough to make decisions about the ACA [24]. In answering four knowledge-based questions, only 3% answered all of them correctly. Future studies should consider enrollment trends and health outcomes in subsequent years, especially as knowledge about the ACA increases, and participants experience benefits from having insurance.

## Conclusions

Based on evidence that early antiretroviral therapy and suppressed HIV viral load can reduce new HIV infections by at least 96% [21], improving HIV care and treatment has become a recommended HIV prevention strategy [17, 25, 26]. We have demonstrated that insurance enrollment was the most significant predictor of achieving suppressed viral load in this study, even after adjusting for other factors. We have also identified predictors of non-enrollment which will guide future outreach to vulnerable populations. In summary, the ACA provides new insurance access to individuals living with HIV disease, and ADAP-facilitated insurance enrollment improves health outcomes.

## Abbreviations

ACA: Affordable Care Act
ADAP: AIDS Drug Assistance Program
EMR: Electronic Medical Record
FPL: Federal Poverty Level
MSM: Men who have sex with men
OTTR: Organ Transplant Tracking Registry
PDC: Proportion Days Covered
SAS: Statistical Analysis Software
SGRx: ScriptGuideRx
UNMC: University of Nebraska Medical Center
